# Transcriptomic responses to thermal stress in hybrid abalone (*Haliotis discus hannai* ♀ × *H. fulgens* ♂)

**DOI:** 10.3389/fgene.2022.1053674

**Published:** 2022-11-16

**Authors:** Qian Zhang, Jianfang Huang, Chenyu Yang, Jianming Chen, Wei Wang

**Affiliations:** ^1^ Institute of Oceanography, College of Geography and Oceanography, Minjiang University, Fuzhou, China; ^2^ Minjiang University, Fuzhou, China

**Keywords:** hybrid abalone, thermal stress, recovery, protein processing, immune system, DNA replication, energy metabolism

## Abstract

China is the world’s largest abalone producing country. Currently, summer mortality caused by high temperature, is one of the biggest challenges for abalone aquaculture industry. The hybrid abalone (*Haliotis discus hannai* ♀ × *H. fulgens* ♂) was conferred on the “new variety”. It has heterosis for thermal tolerance and has been cultured at large-scale in southern China. In this study, a transcriptome analysis was performed to identify the related genes in this hybrid abalone under thermal stress and recovery stage. Compared to control group (18°C), a total of 75, 2173, 1050, 1349, 2548, 494, and 305 differentially expressed genes (DEGs) were identified at 21°C, 24°C, 27°C, 30°C, 32°C, 29°C, and 26°C, respectively. In this study, 24°C is the critical temperature at which the abalone is subjected to thermal stress. With the temperature rising, the number of stress-responsive genes increased. During the temperature recovering to the optimum, the number of stress-responsive genes decreased gradually. Thus, this hybrid abalone has a rapid response and strong adaptability to the temperature. Under the thermal stress, the abalone triggered a complicated regulatory network including degrading the misfolded proteins, activating immune systems, negative regulation of DNA replication, and activating energy production processes. The more quickly feedback regulation, more abundant energy supply and more powerful immune system might be the underlying mechanisms to fight against thermal stress in this hybrid abalone. These findings could provide clues for exploring the thermal-response mechanisms in abalone. The key genes and pathways would facilitate biomarker identification and thermal-tolerant abalone breeding studies.

## 1 Introduction

Abalone has become an increasingly important aquaculture species with high commercial value. China is the world’s largest abalone producing country, which is resulted from the north-south interregional farming modes (Yu et al., 2021). As the main native species of abalone in China, the Pacific abalone, *Haliotis discus hannai*, is naturally distributed in the coastal areas of Eastern Asia, including Northern China, Korea, and Japan. About 20 years ago, the cultivation area was gradually extended from northern China to subtropical areas such as Fujian province (southern China). The southward migration not only increased the aquaculture area, but also shortened the growth period of Pacific abalone (Yu et al., 2021). Since then, the abalone farming in China has developed rapidly and the production of the abalone in Fujian province accounted for more than 80% of the total national yield (Gao et al., 2020). However, the maximum seawater temperature in Fujian province exceeds 30°C, which is much higher than the optimal temperature (20°C) of Pacific abalone (Kyeong et al., 2020). Although it has been farmed in southern China for decades, high mortality in summer remains the major problem in abalone aquaculture industry (Wu and Zhang, 2016).

In ectothermic animals, environmental temperature would affect the metabolic rates, homeostasis and immune response (Ding et al., 2016; Zhang et al., 2019). In addition, the warmer water could diminish the amount of dissolved oxygen (DO), and suppress the respiration, causing energy imbalance of abalone (Ge et al., 2012). Therefore, exploring the adaption mechanism of the high temperature and accelerating the genetic improvements in thermal tolerance have become the priority of abalone breeding research (Yu et al., 2021). So far, some positive progress has been recorded (He et al., 2017; Zhang et al., 2019; Zhang et al., 2020). Many metabolism- and immune-related pathways like “the protein processing in endoplasmic reticulum (ER)” (Chen et al., 2019), “PI3K-AKT signaling pathway” (Sun et al., 2016), “NF-κB signaling pathway” (Zhang et al., 2014; Zhang et al., 2019; Xiao et al., 2021) and “nucleotide binding and oligomerization domain (NOD) like receptor signaling” (Chen et al., 2019) were suggested to be involved in thermal stress in abalone (De Zoysa et al., 2009; Zhang et al., 2014; Morash and Alter, 2015). It is noteworthy that the heat shock response (HSR) is one of the most common responses to thermal stress. Heat shock proteins (HSPs) act as molecular chaperones, which are classified into six major families: sHSP (small HSP), HSP40, HSP60, HSP70, HSP90, and HSP110, according to their molecular weight (Sreedhar and Csermely 2004; Joly et al., 2010; Tripp-Valdez et al., 2019). HSPs play important roles in protein folding, refolding misfolded proteins and elimination of irreversibly damaged proteins (Huang et al., 2014; Lim et al., 2016; Fang et al., 2019).

Hybridization is an effective way of genetic improvement in aquaculture, which can introduce improved traits to the hybrids (Liang et al., 2014). And the commercial implementation of crossbreeding technology has greatly improved the thermotolerance in abalone (Alter et al., 2017; Xiao et al., 2021). New abalone species that can withstand high temperature were introduced to China. The green abalone, *H. fulgens*, which is naturally distributed along the coasts of subtropical Pacific Ocean of the Americas, has a wide range of temperature adaptability (Diaz et al., 2006). It is more broadly tolerant to high temperature and exhibits a growth optimum in the range 24°C–28°C (Leighton et al., 1981). You et al. (2015) carried out an interspecific hybridization study with introduced *H*. *fulgens* from California (United States) and the main domestic Chinese species of *H. discus hannai*. The hybrid abalone (*H. discus hannai* ♀ × *H. fulgens* ♂) has been proved to have significant heterosis in thermal tolerance and growth rate. This hybrid abalone has been conferred on the “new variety” certification by Ministry of Agriculture, China, in 2018 (Gao et al., 2020). Currently, this new variety was popularized and farmed at large-scale in southern China, which is of a great practical significance to improve the structure of cultured abalone species in China (Chen et al., 2016; Chen N. et al., 2020; Shen et al., 2020). At present, underlying mechanisms of adaptation to thermal stress in abalone remain incompletely defined. This new variety is a good model for analyzing the physiological regulation under thermal stress, and it needs further systematic analysis to accelerate the genetic improvement.

In this study, we used RNA-Seq to analyze the dynamic transcriptome response of this hybrid abalone under thermal stress and recovery stage. Gene expression patterns in seven thermal stress groups were compared with that in control temperature (18°C). The objective of this study is to uncover the differentially expressed genes (DEGs) and pathways in response to thermal stress. Besides, the variation tendency of gene expression will be identified with the change of temperature. Finally, we would summarize a potential regulatory mechanism of this hybrid abalone under thermal stress. The results could provide clues for understanding how abalones cope with the high temperature and highlight potentially important molecular mechanisms for future breeding programs.

## 2 Materials and methods

### 2.1 Thermal stress experiment and sample collection

The experimental abalones were transported from Fujian Zhongxin Yongfeng Industrial Co., Ltd. (Fuzhou, China). About 300 abalones (shell length: 3.24 ± 0.35 cm, shell width: 2.21 ± 0.21 cm) were selected and then acclimated in a thermo-controlled and aerated seawater recirculating system for 7 days. During the acclimation, salinity and temperature were kept at 30‰ and 18°C, respectively. All abalones were fed once daily with seaweed. A thermometer (Fuquan, China) was used for monitoring the temperature during the experiment. A total of 240 abalones were used for thermal stress experiment, which were equally divided into four tanks (70 × 60 × 40 cm). And 60 abalone were set aside as the control group. Based on the previous studies (Liang et al., 2014; Tripp-Valdez et al., 2017; Tripp-Valdez et al., 2018; Tripp-Valdez et al., 2019), the water temperature increased by 3°C/d from the acclimation temperature (18°C) to 21°C, 24°C, 27°C, 30°C, 32°C, 29°C, 26°C, 23°C, and 20°C, and then recovered to 18°C. Abalone was maintained at each temperature for 24 h. DO concentration was measured using a DO meter (Multi 3620 IDS, Germany). Moreover, nine individuals of each group were collected. Gills dissected from abalones were immediately frozen in liquid nitrogen, and finally stored at −80°C. The survival rate was recorded for 10 days.

### 2.2 RNA extraction, library construction, and sequencing

Total RNA from the gills of eight groups (G18, G21, G24, G27, G30, G32, G29, and G26) was extracted using Trizol reagent (Gibco BRL, United States). Total amounts and integrity of RNA were assessed using the RNA Nano 6000 Assay Kit of the Bioanalyzer 2100 system (Agilent Technologies, CA, United States). mRNA was enriched by Oligo (dT) beads. Then, mRNA was fragmented into short fragments using fragmentation buffer and reverse-transcribed into the first-strand cDNA with random primers. After the second-strand cDNA was synthesized, the cDNA fragments were purified with QiaQuick PCR extraction kit (Qiagen, Germany). Then the short cDNA fragments were end repaired, poly (A) added, and ligated to Illumina sequencing adapters. After PCR amplification, the cDNA libraries were sequenced using Illumina NovaSeq 6000 by Novogene (Beijing, China).

### 2.3 *De novo* transcriptome assembly and annotation

Raw reads were filtered by removing ambiguous reads (reads containing adapter, containing N base and low-quality reads) to achieve clean reads by fastp (Chen et al., 2018). All clean data were assembled with Trinity software (version 2.6.6) (Grabherr et al., 2011). On the basis of splicing, Corset (version 4.6) aggregated transcripts into many clusters according to inter-transcriptional shared reads (Davidson and Oshlack, 2014). Then, combined with the expression level of transcripts among different samples and H-Cluster algorithm, transcripts with different expression between samples were separated from the original cluster to establish a new cluster. Finally, each cluster was defined as unigene. Unigenes were used for subsequent annotations. To comprehensively annotate the assemblies, transcripts were compared to several public databases: Nr, Nt, PFAM, KOG, SwissProt, KO, and GO (Evalue<1e-5).

### 2.4 Analysis of differentially expressed genes and functional enrichment

FPKM values (expected number of Fragments Per Kilobase of transcript sequence per Millions base pairs sequenced) were calculated to estimate the gene expression levels. Differential expression analysis was performed using the DESeq2 package in R (version 4.2.0) (R Core Team, 2018). The *p*-values were adjusted using the Benjamini and Hochberg’s approach for controlling the false discovery rate. The genes/transcripts with padj ≤ 0.05 and |log_2_ (fold change)|>1 were considered as DEGs. To illustrate the function of DEGs, GO and KEGG enrichment analysis were performed using Novogene online tools (https://magic.novogene.com). The *p*-values were also adjusted by Benjamini and Hochberg’s approach, and padj<0.05 was used as the threshold. The heatmap of DEGs was visualized by TBtools (Chen C. et al., 2020).

### 2.5 Weighted correlation network analysis and differentially expressed genes cluster analysis

WGCNA was performed using the WGCNA package in R. The package is used to calculate various weighted association analysis, which can be used for gene screening and gene cluster identification. Gene expression values were imported into WGCNA to construct co-expression modules. GO and KEGG pathway enrichment analyses were conducted to analyze the biological functions of the modules.

Moreover, the DEGs in the five comparison groups (G21-vs-G18, G24-vs-G18, G27-vs-G18, G30-vs-G18, and G32-vs-G18) were clustered by STEM (Short Time-series Expression Miner) (http://www.sb.cs.cmu.edu/stem/) with a significant score *p*-value<0.05 to investigate the clustering patterns of DEGs.

### 2.6 Validation of expression profiles using quantitative real-time PCR

Six genes including Cluster-15348.83656 (X-box-binding protein 1-like, XBP1), Cluster-15348.81498 (protein disulfide-isomerase, ERP57), Cluster-15348.81469 (calnexin, CNX), Cluster-15348.82068 (calreticulin, CRT), Cluster-15348.79838 (fructose-bisphosphate aldolase, ALD) and Cluster-15348.80913 (ER chaperone Bip) were selected to evaluate the transcriptome sequencing results by RT-qPCR. About 1 μg total RNA was used to synthesize cDNA by PrimeScript™ RT reagent Kit with gDNA Eraser kit (Takara, Japan) with random primers according to the manufacturer’s instructions. The RT-qPCR reactions were carried out using SYBR (TOYOBO, Osaka, Japan). Gene-specific primers (Supplementary Table S1) were designed using Primer 5.0 (https://www.premierbiosoft.com/), where actin gene was used as the reference gene (Ding et al., 2016). RT-qPCR was conducted with the following conditions: denaturation at 94°C for 2 min, 40 cycles of 94°C for 30 s, annealing temperature for 20 s, 72°C for 30 s. Each sample had four technical replicates. The specificity of the primer set was checked by melting curve analysis. All the expression data were obtained from at least three parallel tests. Then, the relative expression level was calculated with 2^−ΔΔCT^ method, and the RT-qPCR results were compared with the transcriptome data.

## 3 Results

### 3.1 Survival rate and dissolved oxygen concentration

**FIGURE 1:**
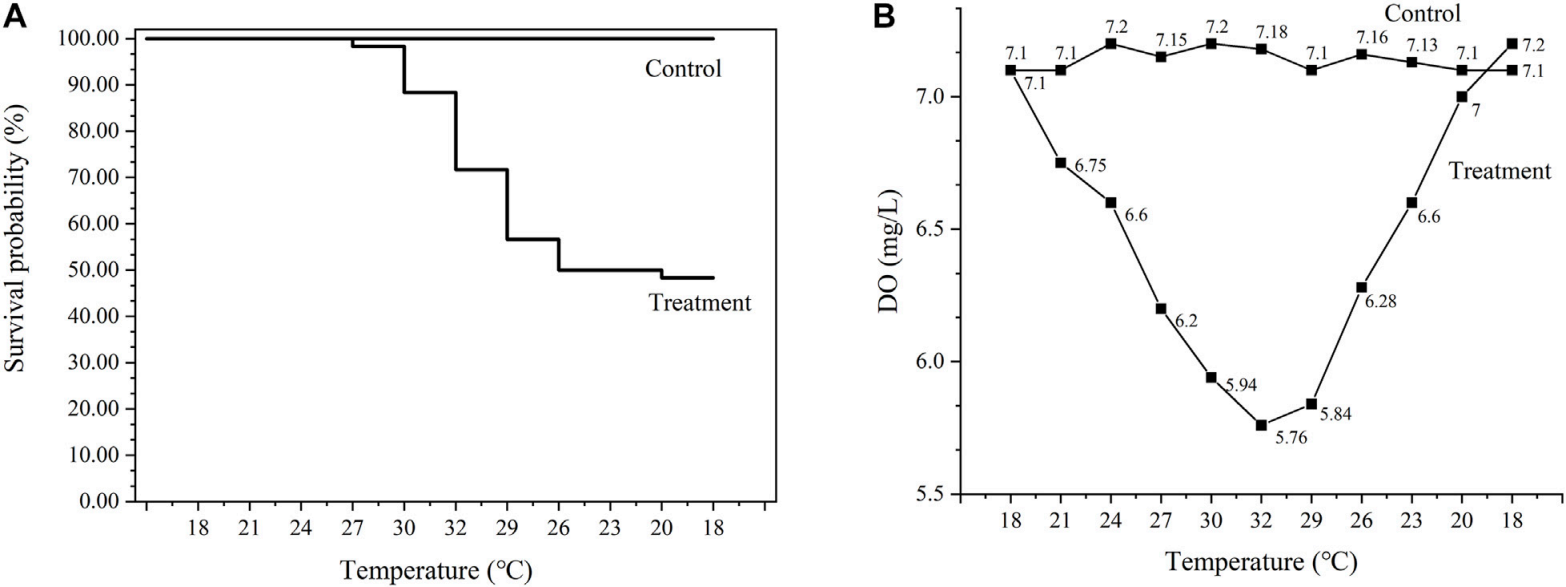
Survival rates of abalone under the thermal stress **(A)** and DO concentration in water **(B)**. Control represents the group at 18°C. Treatment represents thermal stress group.

As shown in Figure 1A, the survival rates of control group and treatment group were 100% and 48.33%, respectively. The initial DO concentration is 7.10 mg/L at 18°C (Figure 1B). With the increase of temperature, DO concentration decreases gradually, and the minimum DO concentration is 5.76 mg/L at 32°C. Subsequently, DO concentration increased gradually as the temperature recovered to 18°C. The DO concentration in control group ranged from 7.10 mg/L to 7.20 mg/L during the experiment.

### 3.2 The basic information of the transcriptome

An overview of sequencing and assembly of the transcriptome is shown in Supplementary Table S2. A total of 522.32 million raw reads and 491.60 million clean reads were obtained, respectively. The raw sequencing data were uploaded to the NCBI with the accession number SRR20075117-SRR20075140. The assembly generated 391688 transcripts with the minimum length of 301 bp and the maximum length of 32 kb. There were 170222 unigenes with an average length of 966 bp and N50 of 1363 bp (Supplementary Table S3). Among 170222 unigenes, 34626, 55584, 12201, 20304, 39770, 39767, and 9103 unigenes can find significant hits in NR, NT, KO, SwissProt, PFAM, GO, and COG database, respectively (Supplementary Table S4). The largest number of unigenes was annotated against NT database.

### 3.3 Identification of differentially expressed genes in response to thermal stress

**FIGURE 2:**
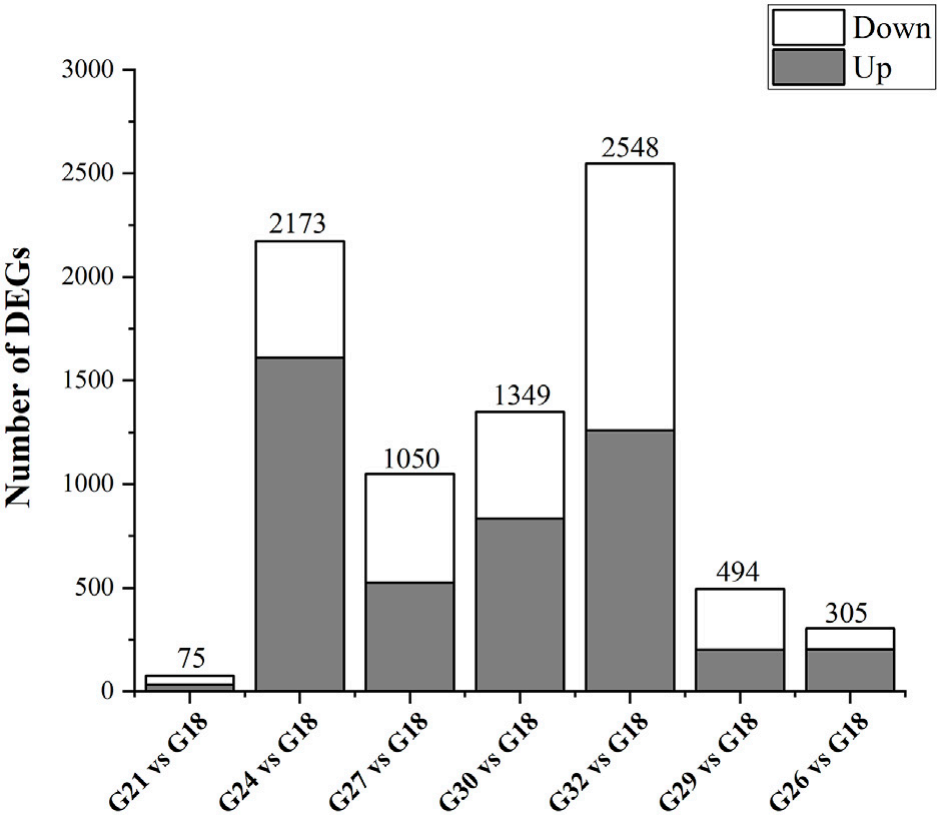
The number of DEGs in response to thermal stress. Up represents highly expressed genes in thermal stress groups (G21, G24, G27, G30, G32, G29, and G26). Down represents highly expressed genes in control group (G18).

Compared to the control group (G18), a total of 75, 2173, 1050, 1349, 2548, 494, and 305 genes showed significantly differential expression patterns in the thermal stress groups (G21, G24, G27, G30, G32, G29, and G26), respectively (Figure 2). G21-vs-G18 has the lowest number of DEGs (75 genes). In G24-vs-G18, the number of DEGs increased significantly. In the range of 27–32°C, the higher temperature, the more DEGs. G32-vs-G18 has the largest number of DEGs, including 1260 up-regulated genes and 1288 down-regulated genes. Subsequently, the number of DEGs decreased rapidly with the temperature recovering to the optimum. The genes selected for validation included XBP1, ERP57, CNX, CRT, ALD, and Bip. The patterns of gene expression indicated by the RT-qPCR and RNA-Seq analyses were consistent (Supplementary Figure S1).

**FIGURE 3:**
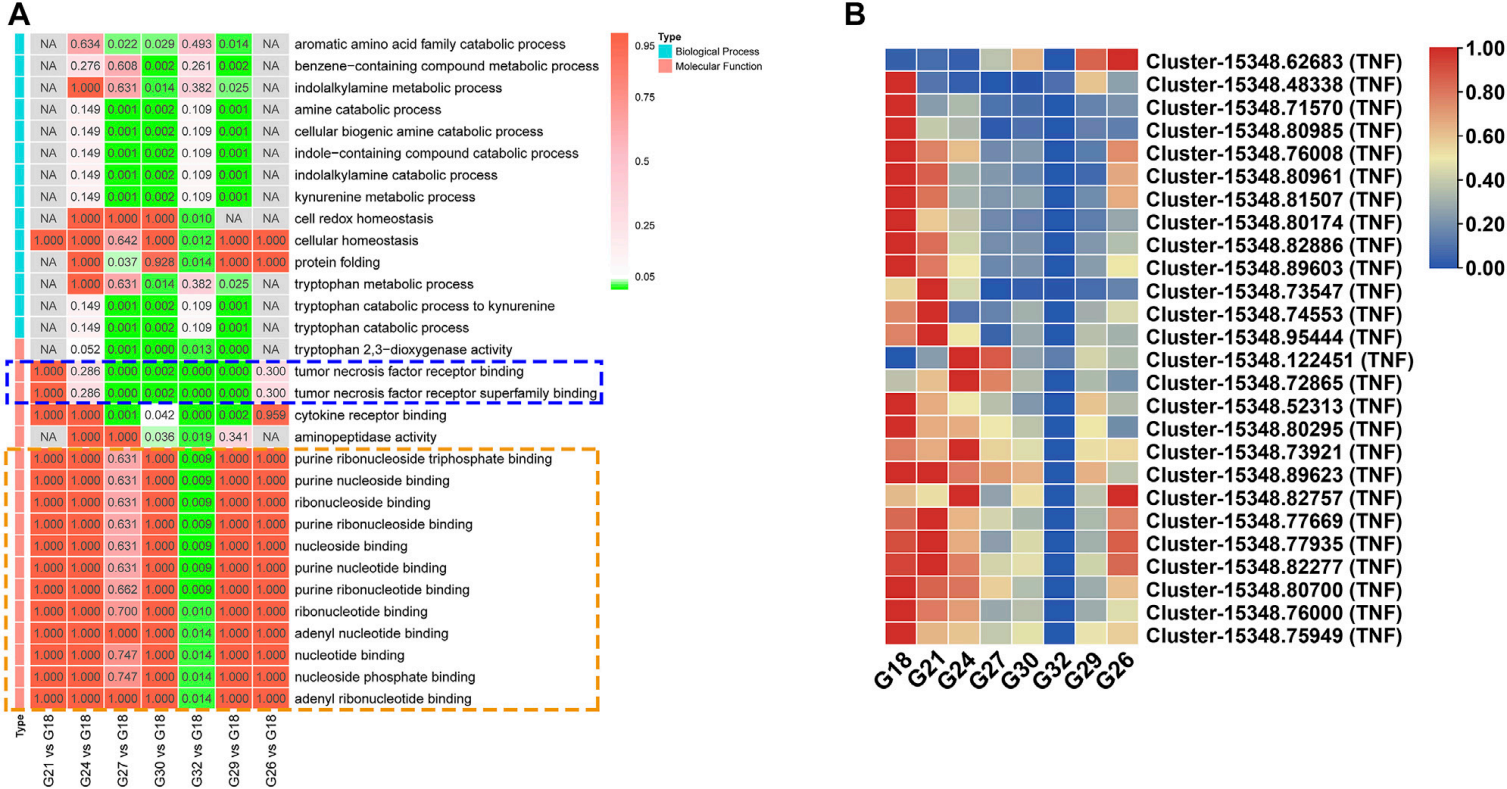
**(A)** Significantly enriched GO terms of the DEGs in seven comparation groups. The terms in blue dotted box are involved in TNF receptor. The terms in orange dotted box are related with purine and nucleotide. **(B)** The expression profiles of DEGs related with “TNF receptor binding” and “TNF receptor superfamily binding”.

GO enrichment analysis of DEGs showed that “tumor necrosis factor (TNF) receptor binding” (GO:0005164) and “TNF receptor superfamily binding” (GO:0032813) were enriched in G27-vs-G18, G30-vs-G18, G32-vs-G18, and G29-vs-G18 (Figure 3A). These two GO terms included 26 TNFs, whose expression levels were negatively correlated with environment temperature (Figure 3B). Twelve GO terms involved in purine and nucleotide were specifically enriched in G32-vs-G18 (Figure 3A). And the gene expression of most DEGs in the above 12 GO terms were down-regulated at 32°C (Supplementary Table S5). In addition, four GO terms involved in tryptophan metabolism including “tryptophan metabolic process” (GO:0006568), “tryptophan catabolic process to kynurenine” (GO:0019441), “tryptophan catabolic process” (GO:0006569) and “tryptophan 2,3-dioxygenase activity” (GO:0004833) were enriched in G27-vs-G18, G30-vs-G18, and G29-vs-G18 (Figure 3A).

**FIGURE 4:**
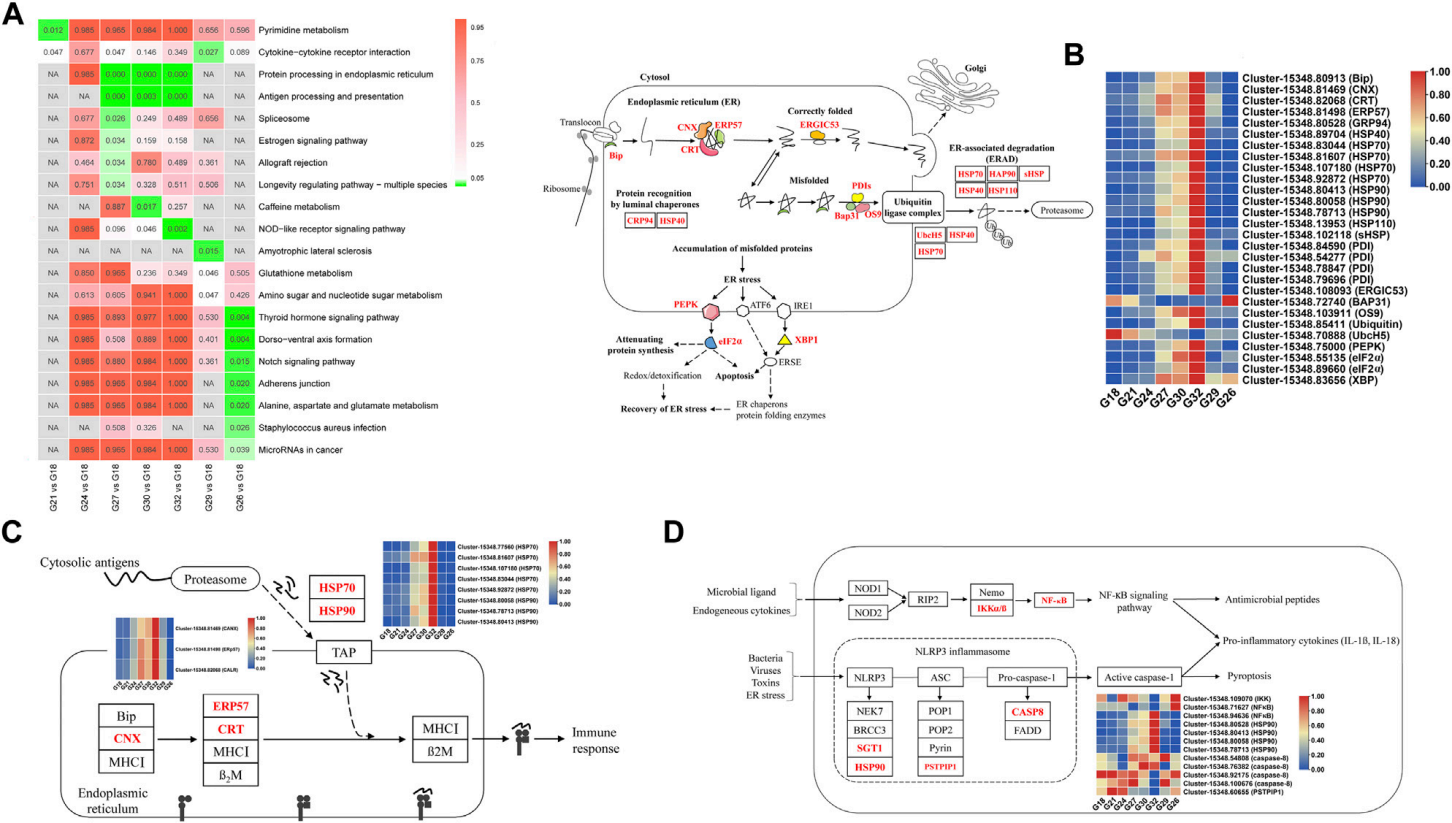
**(A)** Significantly enriched KEGG pathways of the DEGs in seven comparation groups. **(B)** The pathway of “protein processing in ER” and the expression profiles of related DEGs (marked red). **(C)** The pathway of “antigen processing and presentation” and the expression profiles of related DEGs (marked red). **(D)** The pathway of “NOD-like receptor signaling pathway” and the expression profiles of related DEGs (marked red).

KEGG pathway enrichment analysis of DEGs showed “protein processing in ER” (ko04141) was enriched in G27-vs-G18, G30-vs-G18, and G32-vs-G18 (Figure 4A). As for this pathway, the genes related with protein folding including CNX, CRT, ERP57, and ERGIC53 (ERGIC-53 like protein) were up-regulated under thermal stress (Figure 4B). The ER chaperones like Bip, GRP94 (endoplasmin), HSP40 were up-regulated. The DEGs including PDI (protein disulfide-isomerase), BAP31 (B-cell receptor-associated protein), OS9 (protein OS-9), HSP70, HSP90, HSP40, HSP110, sHSP, and UbcH5 (ubiquitin-conjugating enzyme) were involved in misfolded proteins (Figure 4B). Except for BAP31 and UbcH5, other DEGs were activated under thermal stress (Figure 4B). In addition, ER stress triggers two protective cellular pathways. PEPK (translation initiation factor 2-alpha kinase 3) and eIF2α (translation initiation factor 2α) in PERK-ATF4 signaling pathway and XBP1 in IRE1α-XBP1 signaling pathway were all up-regulated in thermal stress groups (Figure 4B).

Several immune pathways were strongly associated with thermal response, especially under extreme thermal stress. “Antigen processing and presentation” (ko04612) was enriched in G27-vs-G18, G30-vs-G18, and G32-vs-G18 (Figure 4A). The DEGs in this pathway were CNX, ERP57, CRT, HSP70 and HSP90, and the expression levels were the highest at 32°C (Figure 4C). “NOD-like receptor signaling pathway” (ko04621) was enriched in G32-vs-G18 (Figure 4A). The DEGs related with initiating “NF-κB signalling pathway” were IKK (protein kinase domain protein) and NF-κB (nuclear factor NF-κB p105 subunit), and they were down-regulated at 32°C (Figure 4D). SGT1 (suppressor of G2 allele of SKP1), HSP90, caspase-8 and PSTPIP1 (proline-serine-threonine phosphatase-interacting protein 1) were involved in “NLRP3 inflammasome” activation (Figure 4D). Additionally, “glutathione metabolism” (ko00480) was enriched in G29-vs-G18, and several pathways like “Notch signaling pathway” (ko04330) and “alanine, aspartate and glutamate metabolism” (ko00520) were significantly enriched in G26-vs-G18 (Figure 4A).

### 3.4 Weighted correlation network analysis

**FIGURE 5:**
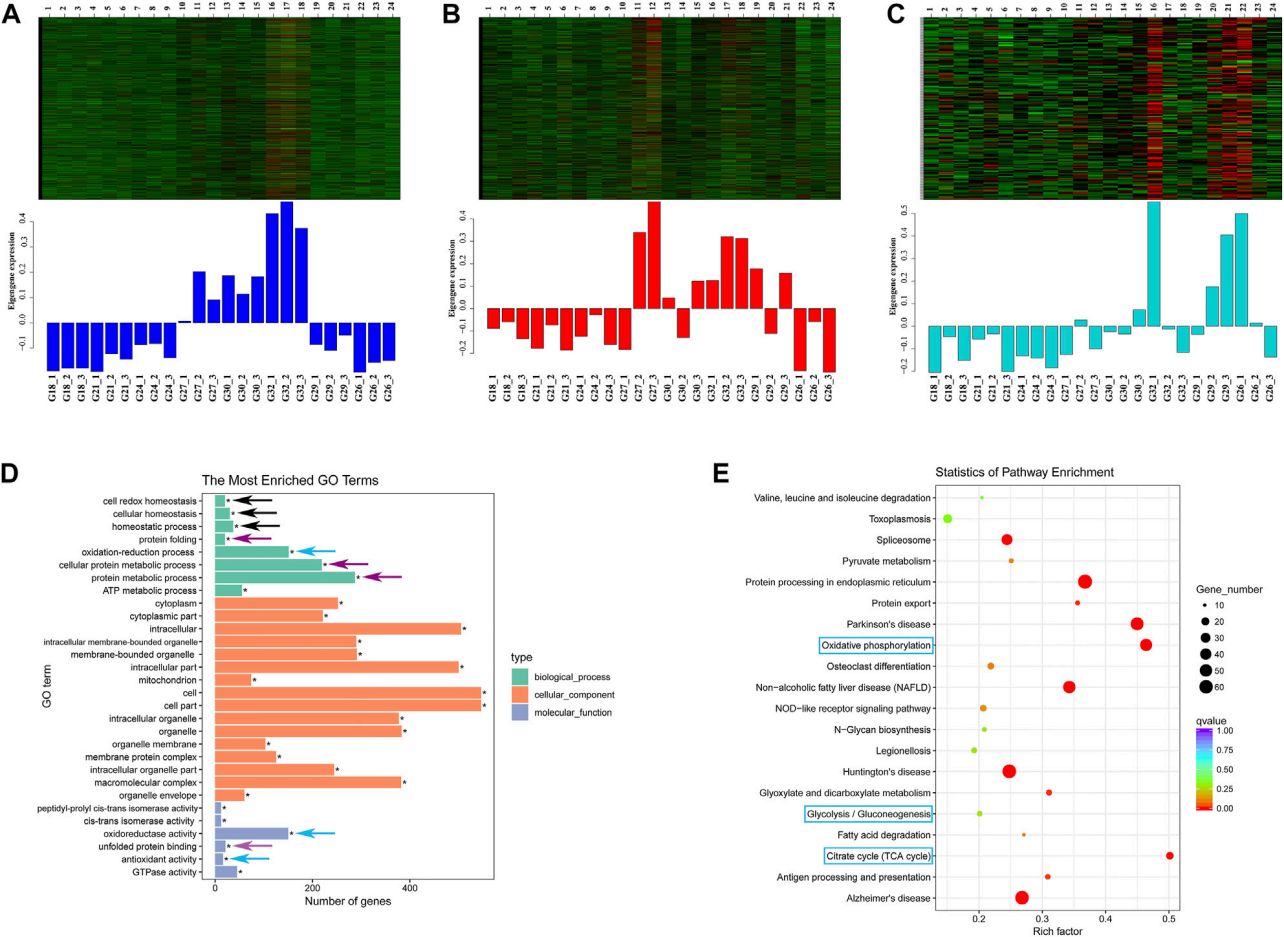
Three modules of blue **(A)** red **(B)** and darkturquoise **(C)** in WGCNA. **(D)** GO terms enrichment analysis of the genes in the above three modules. The black arrows indicate homeostasis; The purple arrows indicate protein metabolic process; The blue arrows indicate oxidation-reduction process. **(E)** KEGG pathways enrichment analysis of the genes in the above three modules. The pathways in blue box are involved in energy-related metabolism.

There were 67 modules in WGCNA (Supplementary Figure S2) and a total of 4060 genes in three modules (blue, red and darkturquoise) were highly expressed in extreme thermal stress groups (G27, G30, G32, and G29) (Figures 5A–C). GO terms analysis of these genes showed that three GO terms related with oxidation-reduction process (“oxidoreductase activity”, GO:0016491; “oxidation-reduction process”, GO:0055114; antioxidant activity, GO:0016209) and three GO terms about homeostasis including “homeostatic process” (GO:0042592), “cell redox homeostasis” GO:0045454), “cellular homeostasis” (GO:0019725) were significantly enriched (Figure 5D). The results showed homeostasis was disrupted in abalone when the temperature was higher than 27°C. Additionally, four GO terms related with protein metabolic process (“protein folding”, GO:0006457; “unfolded protein binding”, GO:0051082; “cellular protein metabolic process”, GO:0044267; “protein metabolic process”, GO:0019538) were also significantly enriched (Figure 5D). The results of KEGG pathway analysis showed that “protein processing in ER”, “antigen processing and presentation”, and “NOD-like receptor signaling pathway” were activated in thermal stress groups. The above pathways were highly consistent with the results in 3.3. Moreover, it is worth mentioning that several energy-related metabolic pathways including “citrate cycle (TCA cycle)” (ko00020), “oxidative phosphorylation” (ko00190) and “glycolysis/gluconeogenesis” (ko00010) were also significantly enriched and the expression of DEGs in these three pathways were all up-regulated (Supplementary Figure S3A,B,C). “Glycolysis” does not require oxygen to produce ATP and ingredients, and it is referred to as anaerobic metabolism. Unlike “glycolysis”, “citrate cycle” and “oxidative phosphorylation” consume oxygen and produce ATP.

### 3.5 Differentially expressed genes cluster analysis with rising temperature

**FIGURE 6:**
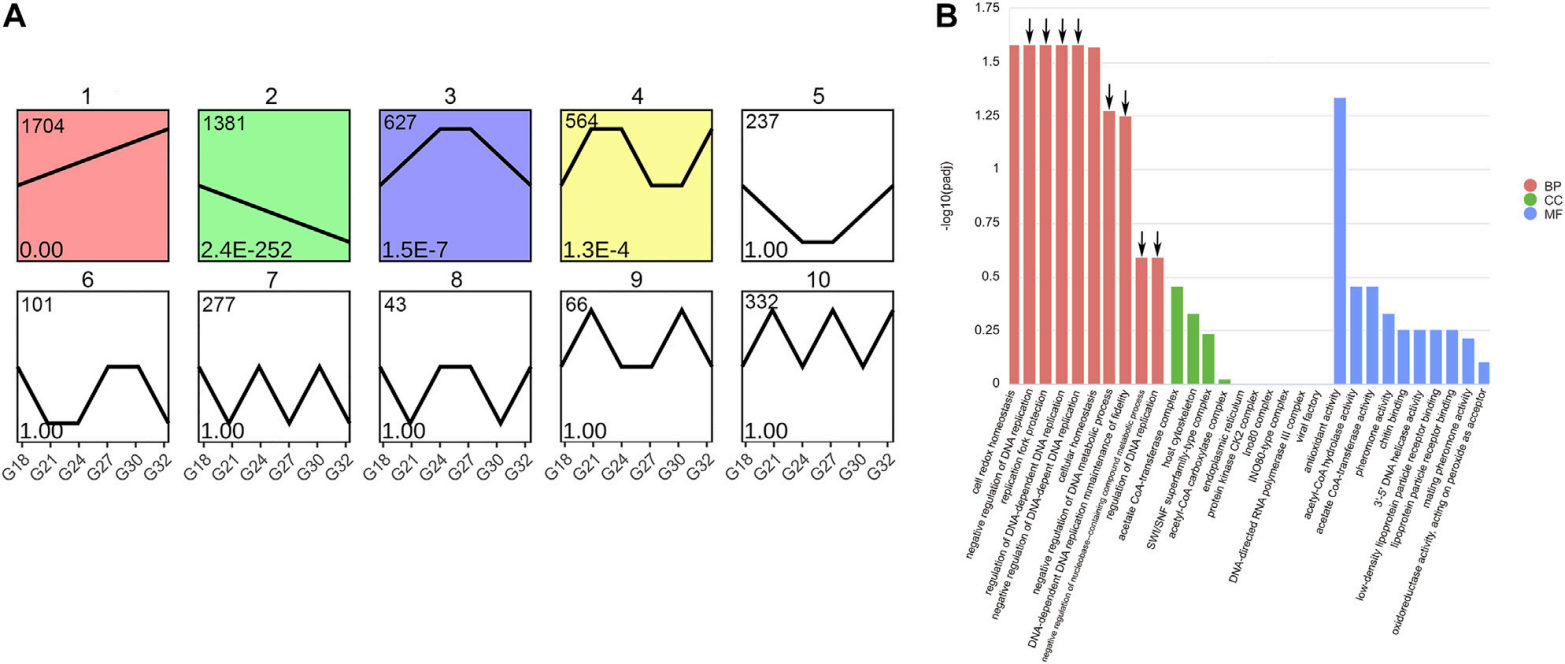
Clusters of DEGs in rising temperature groups (G21, G24, G27, G30, and G32) **(A)** and GO terms enrichment analysis of 1704 DEGs in cluster one **(B)**. The arrows represent the GO terms related with DNA replication. BP represents Biological Process; CC represents Cellular Component; MF represents Molecular Function.

There are a total of 5138 DEGs in the five groups (G21, G24, G27, G30, and G32) with a continuous increase in temperature. The transcriptional profiles of these DEGs were conducted to investigate clustering patterns. As the temperature rising, the expression patterns of the DEGs could be classified into ten clusters. The clusters ranged from 43 to 1704 genes, among which four clusters with significant difference (Figure 6A). The relationship between the expression of these stress-responsive genes and the temperature were not linear. Each cluster showed unique response to the thermal stress. There were 1704 DEGs in the cluster 1, which displayed prolonged gene expression against thermal tress. The results of GO terms analysis of these 1704 DEGs showed “negative regulation of DNA replication” (GO:0008156), “replication fork protection” (GO:0048478), “regulation of DNA-dependent DNA replication” (GO:0090329), “negative regulation of DNA-dependent DNA replication” (GO:2000104), “negative regulation of DNA metabolic process” (GO:0051053), “DNA-dependent DNA replication maintenance of fidelity” (GO:0045005), “negative regulation of nucleobase-containing compound metabolic process” (GO:0045934) and “regulation of DNA replication” (GO:0006275) were significantly enriched (Figure 6B). They were all related with regulation of DNA replication and maintenance of fidelity of DNA replication.

## 4 Discussion

**FIGURE 7:**
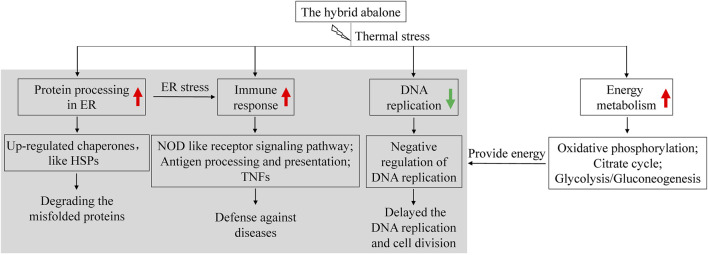
Summary of molecular mechanisms of the abalone in response to thermal stress. The red arrow represents up-regulated gene expression under thermal stress. The green arrow represents down-regulated gene expression under thermal stress.

Thermal stress has been one of the paramount challenges for abalone aquaculture in China. Abalone can trigger the stress response to adapt to harsh environmental conditions. The gill, a primary organ for abalone, plays multiple roles in gas exchange, waste excretion and immune defense (Kyeong et al., 2020). Compared to the control group (G18), numerous transcripts were differentially modulated at 24°C. Therefore, 24°C is likely to be the critical temperature at which the homeostasis of this hybrid abalone is disturbed. At 32°C, the number of DEGs was the largest and decreased as the temperature recovering to the optimum. Therefore, this hybrid variety has a rapid response to the temperature. Under the thermal stress, the molecular mechanisms of this hybrid abalone included degrading the misfolded proteins, activating immune systems, down-regulation of DNA replication, and activating energy production processes (Figure 7).

“Protein processing in ER” was strongly activated under thermal stress, which is consistent with the previous findings in abalone (Chen et al., 2018; Xiao et al., 2021) and other species (Huang et al., 2018; Cui et al., 2019; Srikanth et al., 2019). ER is a subcellular organelle where proteins are folded. Correctly folded proteins are packaged into transport vesicles that shuttle them to the Golgi complex. Misfolded proteins are retained within the ER lumen in complex with molecular chaperones (Maattanen et al., 2010; Stolz and Wolf, 2010). Accumulation of misfolded proteins causes ER stress and activates the induction of chaperones, degradation of misfolded proteins and attenuation of protein translation to restore normal ER function (Welihinda et al., 1999; Malhotra and Kaufman, 2007; Naidoo, 2009; Stolz and Wolf, 2010). Furthermore, ER stress triggers two protective cellular responses by initiating PERK-ATF4 and IRE1α-XBP1 signaling pathway (Menu et al., 2012; Blander, 2014). However, excessive and prolonged stress will lead to a maladaptive response and apoptosis (Naidoo, 2009). In this study, ER chaperones such as Bip, ERP57, GRP94, HSP40, HSP70, HSP90, PDI, and OS9 were significantly up-regulated in high temperature groups, which indicated thermal stress led to an increase of the misfolded proteins. The induction of HSPs upon thermal stress is widely reported in mollusks and the tolerant line showed higher expression levels (Shiel et al., 2015; Kim et al., 2017; Chen et al., 2019). In this study, HSPs were strongly affected by thermal stress, which could act as indicators to identify the thermal stress of abalone and monitor intracellular thermal stress conditions (Wan et al., 2012; Lim et al., 2016).

Tolerance to disease in abalone varies greatly at different temperatures (Vilchis et al., 2005; Liang et al., 2014). In the present study, several immune pathways in abalone were strongly associated with thermal response. TNFs play important roles in apoptosis and immune responses (De Zoysa et al., 2009; Ward-Kavanagh et al., 2016). In this study, TNFs in abalone were down-regulated under thermal stress, and this finding is rarely reported in previous study. “NOD like receptor signaling pathway” was significantly changed, which was agreement with previous studies of other abalone species (Chen et al., 2019; Xiao et al., 2021). In addition, “antigen processing and presentation” was up-regulated under thermal stress, which is similar to the findings of Xiao et al. (2021). The transporter associated with antigen processing is essential for immunogenic peptide delivery from the cytosol into the lumen of the ER. Subsequently, pathogen-infected or malignantly transformed cells can be eliminated (Abele and Tampé, 2004; Trombetta and Mellman, 2005). Thus, we suggest that abalone might be more susceptible to pathogens under high temperature. In the previous study, elevated temperature increased the susceptibility to withering syndrome (WS) in black abalone (*H. cracherodii*), and mass mortality caused by WS is more likely to occur at warm water temperature (Ben-Horin et al., 2013). Overall, our results demonstrate that the immune system of abalone under high temperature is activated. And the high mortality of abalone in summer might be caused by the multiple effects such as high temperature and diseases.

Twelve enriched GO terms in G32-vs-G18 were related with purine and nucleotide binding and most genes were obviously suppressed at high temperature (Figure 3A). Interestingly, in the cluster analysis, eight significantly enriched GO terms of prolonged up-regulated genes were related with negative regulation of DNA replication and maintenance of fidelity of DNA replication (Figure 6B). Our work reveals that cell cycle and DNA replication of abalone were suppressed under thermal stress. This finding has not been reported in previous studies and it maybe a unique responding mechanism of this hybrid abalone. In stressful conditions, cells would delay the DNA replication and cell division in favor of cytoprotective functions and maintenance of fidelity (Jonas, et al., 2013). Thus, negative regulation of DNA replication might be an important protection strategy, reducing the heat-damage.

In general, temperature is an important modulator of the metabolic rate in marine animals. As temperature increases, the rate of physiological reactions increases, which requires more energy and oxygen (Morash and Alter, 2015; Xu et al., 2020; Zhang et al., 2022). However, the DO content decreases about 1.50 mg/L from 18°C to 32°C. Therefore, the high temperature in summer not only increases the demand for oxygen of abalone, but also reduces DO content in water (Gao et al., 2020). How does the abalone cope with this conflict? The results showed thermal stress initiates both aerobic and anaerobic metabolism to produce energy. On the one hand, “citrate cycle” and “oxidative phosphorylation” were activated, which is consistent with Xu et al. (2020). On the other hand, anaerobic metabolism was also activated. “Glycolysis/gluconeogenesis” was significantly up-regulated in the high temperature environment, producing more energy and ingredients for metabolism. Therefore, short-term acute thermal stress could activate both aerobic to anaerobic metabolisms to produce energy in this hybrid abalone.

## 5 Conclusion

In summary, this study provides preliminary insights into the molecular mechanism of the hybrid abalone under thermal stress. In this study, 24°C is likely to be the critical temperature at which abalones are subjected to thermal stress. The results confirmed that this hybrid abalone was more tolerant to high temperature than its female parent. The higher the temperature, the more stress-responsive genes are mobilized. During the temperature recovering to the optimum, the number of stress-responsive genes decreased gradually. Thus, this hybrid abalone has a rapid response and strong adaptability to the temperature. Under thermal stress, the abalone triggered a complicated regulatory network, including degrading the misfolded proteins, activating immune systems and negative regulation of DNA replication. Besides, aerobic and anaerobic respiration rates were both increased to produce energy and ingredients. The more quickly feedback regulation, more abundant energy supply and more powerful immune system might be the effective underlying mechanisms to cope with thermal stress of this hybrid abalone. Overall, these findings could provide some suggestions for further studies to understand the molecular basis of thermal adaptation in mollusks. The key genes and pathways would provide fundamental information for breeding the thermal-tolerant abalone.

## Data Availability

The datasets presented in this study can be found in online repositories. The names of the repository/repositories and accession number(s) can be found below: We have uploaded the raw data to the GenBank (Accession number: PRJNA853707).

## Supplementary material

Supplementary Figure S1 Validation of expression profiles of DEGs in transcriptome. **(A)** XBP1; **(B)** ERP57; **(C)** CNX; **(D)** CRT; **(E)** ALD; **(F)** Bip. These data were expressed as the mean ± SD relative to the reference gene (actin). Histogram represents relative expression detected by RT-qPCR. Line graph represents FPKM in transcriptome.

Supplementary Figure S2 A hierarchical clustering dendrogram in WGCNA.

Supplementary Figure S3 The heatmap of DEGs related with energy metabolism. **(A)** The DEGs in the pathway of “citrate cycle (TCA cycle)”; **(B)** The DEGs in the pathway of “oxidative phosphorylation”; **(C)** The DEGs in the pathway of “glycolysis/gluconeogenesis”.

Supplementary TABLE S1 Primer sequences and annealing temperature used for RT-qPCR.

Supplementary TABLE S2 Summary of the transcriptome data from abalone.

Supplementary TABLE S3 Trinity assembly statistics.

Supplementary TABLE S4 Annotation statistics.

Supplementary TABLE S5 Gene expression of all DEGs involved in 12 GO terms about purine and nucleotide.
