# Supplementary figures and images for: Transcriptomic responses to thermal stress in hybrid abalone (Haliotis discus hannai ♀ × H. fulgens ♂)
Source: Front Genet. 2022 Nov 16;13:1053674. doi: 10.3389/fgene.2022.1053674 (PMC9709276; doi:10.3389/fgene.2022.1053674)

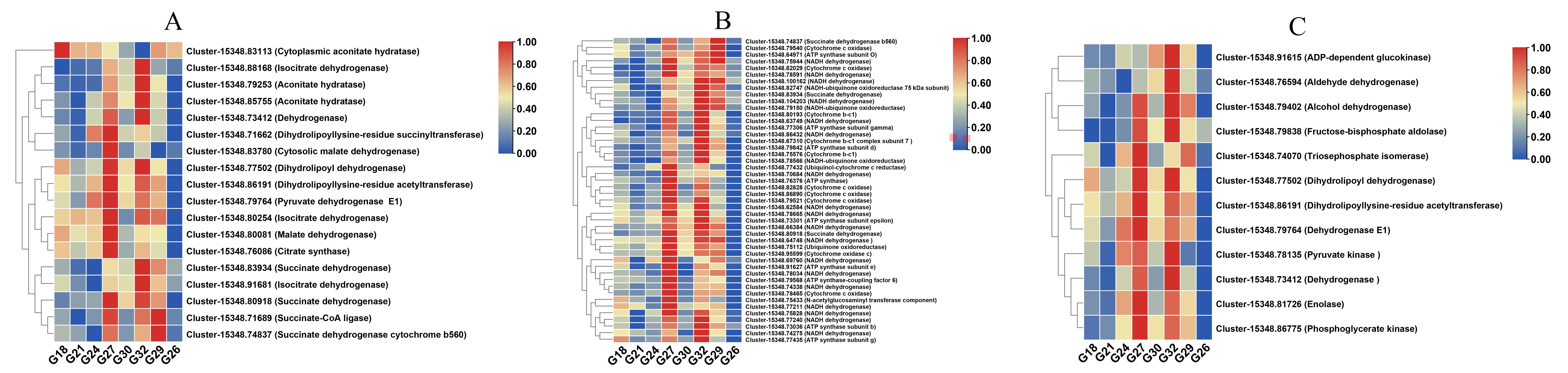

Supplement: Supplementary file 1 [file Image3.JPEG]

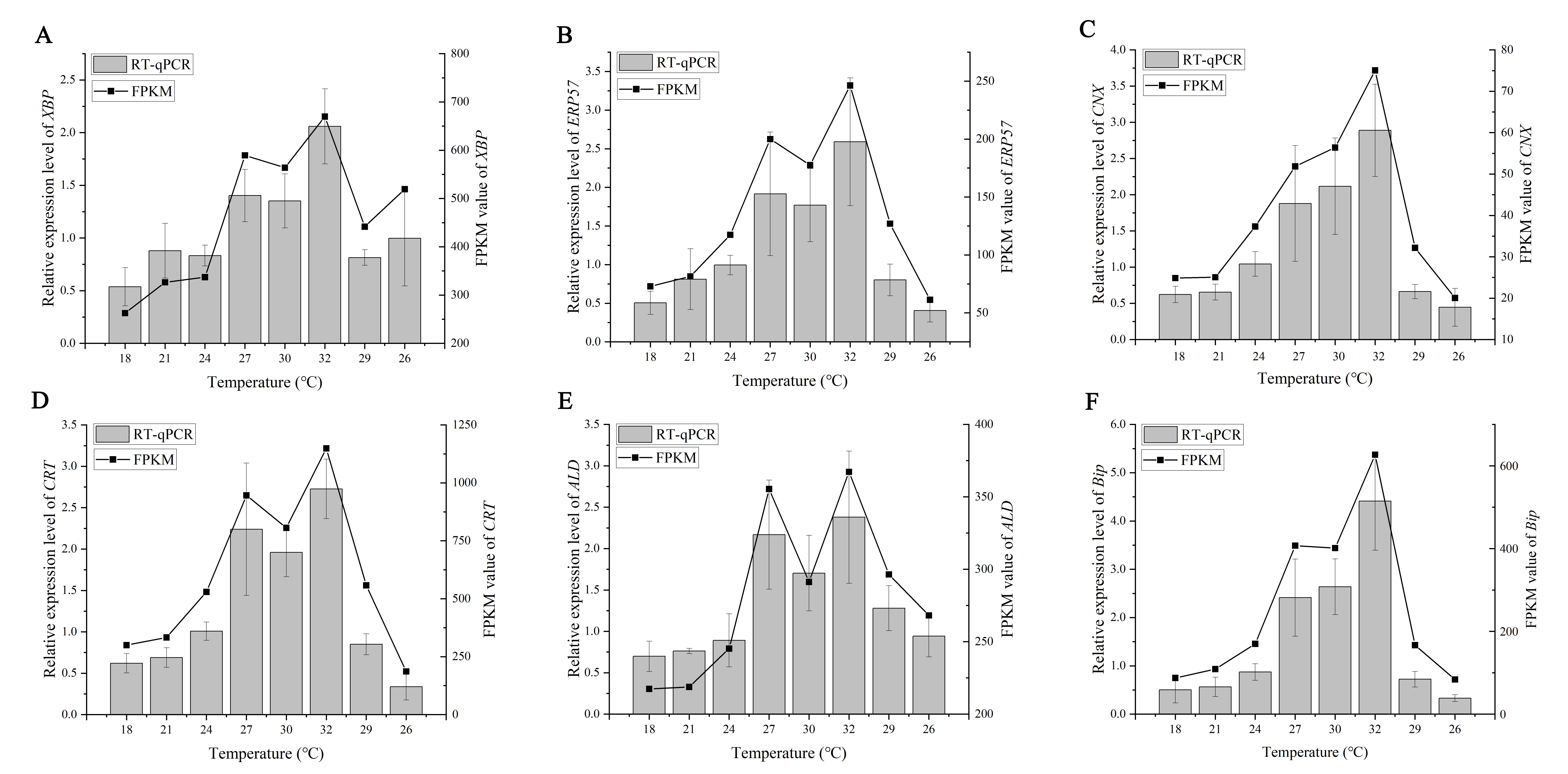

Supplement: Supplementary file 2 [file Image1.JPEG]

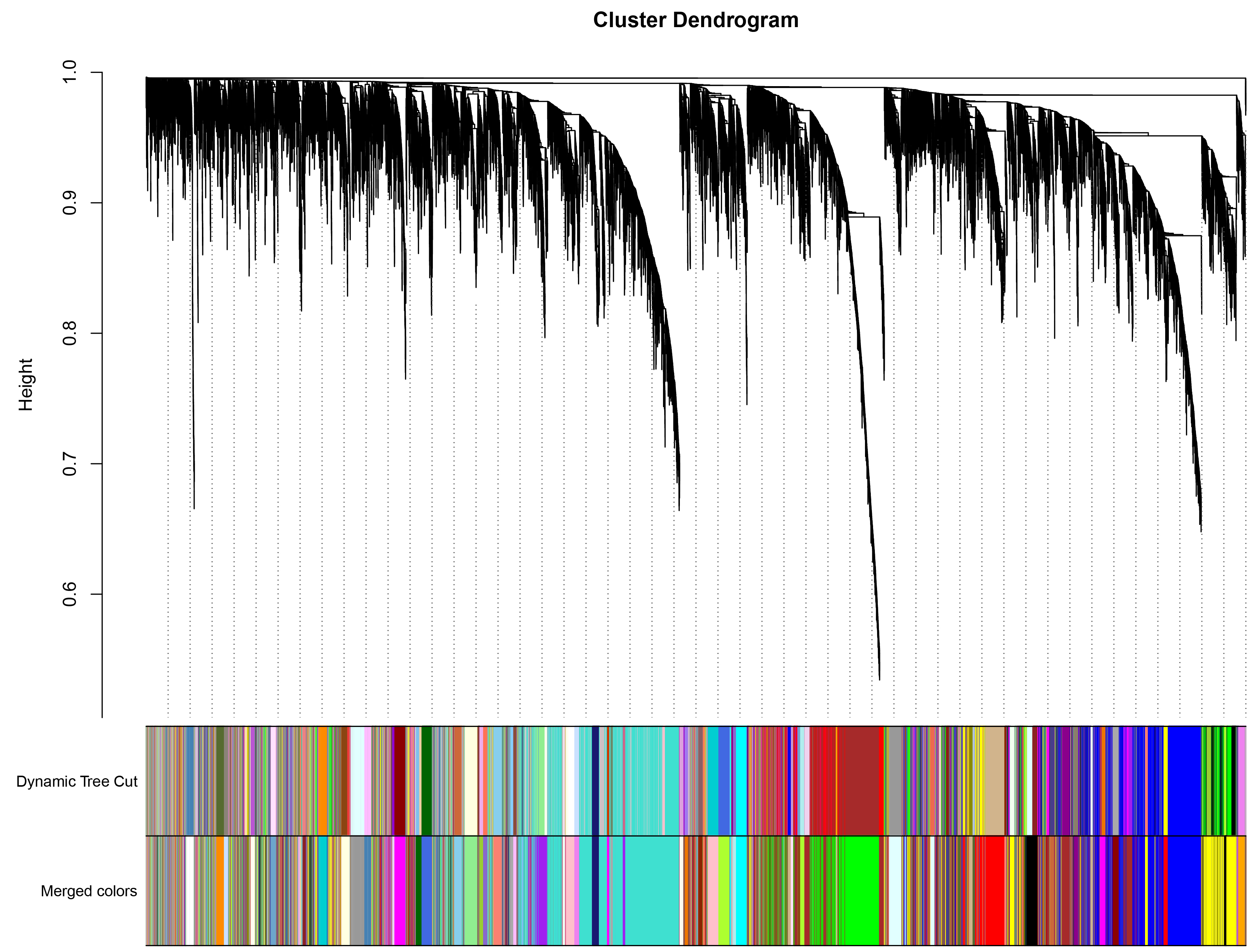

Supplement: Supplementary file 3 [file Image2.JPEG]
