# Supplementary material for: Transcriptomic responses to thermal stress in hybrid abalone (Haliotis discus hannai ♀ × H. fulgens ♂)
Source: Front Genet. 2022 Nov 16;13:1053674. doi: 10.3389/fgene.2022.1053674 (PMC9709276; doi:10.3389/fgene.2022.1053674)
Supplement: Supplementary file 4 [file DataSheet1.docx]

**TABLE S1 |** Primer sequences and annealing temperature used for RT-qPCR.

| **Gene­­** | **Gene ID** | **Forward primer (5' to 3')** | **Reverse primer (5' to 3')** | **Product size (bp)** | **Tm (℃)** |
| --- | --- | --- | --- | --- | --- |
| XBP1 | Cluster-15348.83656 | GGTGGCTTCGTCTGTCA | GGGAGAATGCTGCGTAGTG | 144 | 55 |
| ERP57 | Cluster-15348.81498 | CCCTCCATCCTGTATTTC | GACTGTCTACTTCGCCATC | 135 | 51 |
| CNX | Cluster-15348.81469 | TGTTACTGTAGGTGGTGTTCG | ACTGGTGGAGGCACTGAT | 137 | 55 |
| CRT | Cluster-15348.82068 | GCAGCCTTTGCGGTTGAA | ATGAGGGCGGTTGCGATT | 125 | 57 |
| ALD | Cluster-15348.79838 | CCGATACACTGGGAACAA | CATAAATGAGGGATGGAAAA | 162 | 50 |
| Bip | Cluster-15348.80913 | CAGAGGGTTATGGAGGATT | CAGCACCGTATGCGACAG | 169 | 53 |
| Actin | XM_046516532.2 | ACGAAGATGTTGCTGCGTTGGTT | TCGATGGGGTACTTGAGGGTGAG | 211 | 58 |

**TABLE S2 |** Summary of the transcriptome data from abalone.

| **Sample** | **Raw reads** | **Clean reads** | **Q20 (%)** | **Q30 (%)** |
| --- | --- | --- | --- | --- |
| G18-1 | 22008950 | 20906716 | 98.08 | 94.34 |
| G18-2 | 22671502 | 21326873 | 97.96 | 94.08 |
| G18-3 | 21349882 | 20223684 | 97.94 | 94.05 |
| G21-1 | 21411703 | 20168966 | 98.03 | 94.25 |
| G21-2 | 21334748 | 20161602 | 97.96 | 94.07 |
| G21-3 | 21492535 | 20156170 | 98.08 | 94.27 |
| G24-1 | 21494865 | 20549763 | 98.07 | 94.27 |
| G24-2 | 21356625 | 20641073 | 97.89 | 93.92 |
| G24-3 | 21552480 | 20344287 | 97.89 | 93.91 |
| G27-1 | 23555187 | 22480873 | 97.95 | 94.05 |
| G27-2 | 20430588 | 18989197 | 98.04 | 94.27 |
| G27-3 | 24389628 | 22605502 | 97.92 | 93.96 |
| G30_1 | 21217465 | 19774401 | 98.11 | 94.46 |
| G30_2 | 20151589 | 18986317 | 98.06 | 94.36 |
| G30_3 | 23494154 | 22095122 | 98.02 | 94.28 |
| G32_1 | 20377530 | 19206180 | 97.85 | 93.80 |
| G32_2 | 20380388 | 19115452 | 98.00 | 94.20 |
| G32_3 | 23732218 | 22256977 | 97.40 | 92.89 |
| G29_1 | 21291041 | 19655027 | 98.03 | 94.22 |
| G29_2 | 20351343 | 19083809 | 97.94 | 94.07 |
| G29_3 | 21501459 | 20074637 | 98.03 | 94.22 |
| G26_1 | 21908557 | 20518798 | 97.92 | 93.98 |
| G26_2 | 22652568 | 21522789 | 97.91 | 93.99 |
| G26_3 | 22215794 | 20757294 | 98.05 | 94.29 |

**TABLE S3 |** Trinity assembly statistics.

| **Type** | **Min length (bp)** | **Mean length (bp)** | **Median length (bp)** | **Max length (bp)** | **N50 (bp)** | **N90 (bp)** | **Total nucleotides** | **Number** |
| --- | --- | --- | --- | --- | --- | --- | --- | --- |
| Transcript | 301 | 1116 | 685 | 32031 | 1633 | 470 | 437243678 | 391688 |
| Unigene | 301 | 966 | 576 | 32031 | 1363 | 414 | 164451489 | 170222 |

**TABLE S4 |** Annotation statistics.

| **Annotated in database** | **Number of unigenes** |
| --- | --- |
| NR | 34626 |
| NT | 55584 |
| KO | 12201 |
| SwissProt | 20304 |
| PFAM | 39770 |
| GO | 39767 |
| KOG | 9103 |
| At least one database | 90635 |
| Total unigenes | 170222 |

**TABLE S5 |** Gene expression of all DEGs involved in 12 GO terms about purine and nucleotide.

| **Gene ID** | **G18 FPKM** | **G21 FPKM** | **G24 FPKM** | **G27 FPKM** | **G30 FPKM** | **G32 FPKM** | **G29 FPKM** | **G26 FPKM** |
| --- | --- | --- | --- | --- | --- | --- | --- | --- |
| Cluster-15348.100497 | 13.88 | 16.17 | 18.10 | 37.28 | 36.54 | 78.97 | 32.39 | 20.10 |
| Cluster-15348.100676 | 3.61 | 4.45 | 5.27 | 6.16 | 3.25 | 0.94 | 6.19 | 2.91 |
| Cluster-15348.101806 | 13.82 | 15.10 | 8.44 | 7.50 | 3.89 | 1.66 | 1.77 | 6.48 |
| Cluster-15348.102877 | 33.06 | 29.84 | 40.53 | 40.30 | 23.29 | 12.02 | 30.25 | 33.27 |
| Cluster-15348.103760 | 3.96 | 4.00 | 3.90 | 8.24 | 7.98 | 11.92 | 5.44 | 4.03 |
| Cluster-15348.104768 | 2.55 | 2.98 | 6.15 | 4.04 | 7.42 | 14.96 | 2.71 | 3.74 |
| Cluster-15348.104952 | 3.88 | 3.95 | 4.57 | 7.58 | 10.12 | 15.72 | 4.93 | 4.29 |
| Cluster-15348.106524 | 7.04 | 3.99 | 3.31 | 1.61 | 3.04 | 1.51 | 3.30 | 4.00 |
| Cluster-15348.109070 | 3.36 | 0.50 | 4.24 | 3.34 | 2.00 | 0.22 | 2.62 | 4.63 |
| Cluster-15348.109298 | 1.79 | 3.08 | 4.92 | 10.69 | 19.16 | 55.16 | 5.71 | 3.48 |
| Cluster-15348.110897 | 2.15 | 2.42 | 3.42 | 4.43 | 4.68 | 6.58 | 5.99 | 3.69 |
| Cluster-15348.111738 | 0.78 | 0.97 | 0.74 | 0.26 | 0.71 | 0.19 | 0.44 | 0.52 |
| Cluster-15348.114290 | 2.47 | 2.56 | 2.64 | 3.26 | 5.06 | 8.30 | 3.22 | 1.71 |
| Cluster-15348.114690 | 17.25 | 16.01 | 7.79 | 9.02 | 2.51 | 1.72 | 2.15 | 8.16 |
| Cluster-15348.117378 | 0.01 | 2.98 | 1.47 | 0.00 | 2.96 | 3.08 | 4.94 | 9.80 |
| Cluster-15348.118672 | 0.05 | 0.51 | 0.26 | 0.23 | 0.09 | 3.86 | 0.07 | 0.00 |
| Cluster-15348.120066 | 0.95 | 0.96 | 0.94 | 2.73 | 2.31 | 5.12 | 1.83 | 1.39 |
| Cluster-15348.120884 | 0.29 | 0.36 | 1.34 | 0.64 | 3.87 | 2.88 | 1.77 | 0.80 |
| Cluster-15348.25527 | 2.34 | 1.20 | 0.50 | 0.17 | 0.20 | 0.00 | 0.00 | 0.00 |
| Cluster-15348.30781 | 0.00 | 0.04 | 0.34 | 0.12 | 0.61 | 0.59 | 0.28 | 0.14 |
| Cluster-15348.31892 | 0.45 | 7.25 | 0.54 | 2.28 | 5.19 | 6.59 | 1.57 | 0.57 |
| Cluster-15348.31974 | 3.12 | 1.44 | 0.57 | 0.43 | 0.25 | 0.03 | 0.00 | 0.00 |
| Cluster-15348.32016 | 0.72 | 19.57 | 23.79 | 109.07 | 40.37 | 17.60 | 26.54 | 60.58 |
| Cluster-15348.32845 | 3.06 | 3.47 | 3.49 | 5.43 | 5.07 | 12.08 | 5.66 | 3.76 |
| Cluster-15348.37516 | 10.09 | 7.91 | 0.88 | 18.07 | 13.02 | 109.28 | 3.04 | 47.52 |
| Cluster-15348.45735 | 0.89 | 1.42 | 2.45 | 0.18 | 0.33 | 0.00 | 0.41 | 1.30 |
| Cluster-15348.46021 | 4.87 | 2.86 | 2.68 | 2.74 | 2.06 | 1.23 | 2.82 | 2.79 |
| Cluster-15348.46886 | 0.06 | 0.09 | 0.33 | 0.34 | 1.56 | 2.61 | 0.32 | 0.28 |
| Cluster-15348.46931 | 3.35 | 2.85 | 2.44 | 1.60 | 2.62 | 0.84 | 2.56 | 2.10 |
| Cluster-15348.47181 | 0.12 | 0.40 | 0.58 | 0.19 | 1.46 | 2.36 | 0.29 | 0.39 |
| Cluster-15348.47195 | 5.00 | 0.07 | 0.23 | 0.14 | 0.07 | 0.12 | 0.06 | 1.31 |
| Cluster-15348.47715 | 24.77 | 17.93 | 13.12 | 1.51 | 5.99 | 0.28 | 3.38 | 5.46 |
| Cluster-15348.47926 | 1.88 | 0.30 | 1.54 | 0.73 | 0.90 | 0.11 | 1.78 | 1.93 |
| Cluster-15348.47993 | 0.66 | 0.60 | 0.90 | 1.47 | 1.40 | 2.92 | 1.50 | 0.78 |
| Cluster-15348.49976 | 72.92 | 87.98 | 41.83 | 15.79 | 40.45 | 3.49 | 18.90 | 29.04 |
| Cluster-15348.50124 | 14.75 | 8.82 | 8.43 | 183.66 | 27.42 | 217.13 | 113.64 | 317.06 |
| Cluster-15348.51190 | 2.43 | 2.23 | 2.35 | 3.45 | 4.85 | 7.57 | 2.95 | 2.07 |
| Cluster-15348.52423 | 1.92 | 2.57 | 2.94 | 4.76 | 4.08 | 8.24 | 4.43 | 1.95 |
| Cluster-15348.52569 | 2.28 | 3.08 | 2.05 | 1.26 | 1.88 | 0.45 | 1.42 | 1.67 |
| Cluster-15348.53233 | 17.95 | 9.89 | 8.42 | 3.98 | 2.37 | 1.09 | 1.32 | 2.96 |
| Cluster-15348.53908 | 9.67 | 4.75 | 3.86 | 3.34 | 2.09 | 0.81 | 1.93 | 2.86 |
| Cluster-15348.53983 | 3.06 | 2.71 | 3.73 | 7.22 | 7.97 | 23.64 | 5.86 | 5.89 |
| Cluster-15348.55135 | 1.76 | 2.00 | 2.07 | 4.72 | 7.10 | 8.05 | 1.95 | 3.39 |
| Cluster-15348.60198 | 43.47 | 29.46 | 23.24 | 14.16 | 13.88 | 11.96 | 19.79 | 35.31 |
| Cluster-15348.61577 | 43.65 | 41.88 | 70.87 | 210.43 | 260.44 | 433.26 | 50.37 | 35.66 |
| Cluster-15348.62200 | 1.10 | 0.42 | 1.95 | 0.74 | 1.30 | 0.13 | 0.51 | 0.30 |
| Cluster-15348.62369 | 38.53 | 22.69 | 20.05 | 10.71 | 12.36 | 7.13 | 12.76 | 36.28 |
| Cluster-15348.63016 | 6.01 | 8.58 | 7.76 | 10.52 | 15.18 | 45.37 | 11.88 | 33.95 |
| Cluster-15348.63360 | 8.91 | 14.39 | 10.28 | 2.63 | 5.85 | 0.20 | 0.17 | 10.79 |
| Cluster-15348.64477 | 34.41 | 16.20 | 17.15 | 13.86 | 13.24 | 7.87 | 11.60 | 25.83 |
| Cluster-15348.64669 | 5.10 | 5.85 | 5.62 | 10.85 | 11.08 | 22.15 | 11.66 | 16.44 |
| Cluster-15348.66321 | 25.55 | 6.93 | 5.08 | 3.22 | 7.12 | 1.74 | 2.36 | 18.83 |
| Cluster-15348.68504 | 3.27 | 4.10 | 2.69 | 2.33 | 2.66 | 0.92 | 3.86 | 3.01 |
| Cluster-15348.68774 | 12.48 | 10.30 | 22.98 | 13.69 | 7.18 | 2.66 | 6.23 | 15.10 |
| Cluster-15348.69009 | 22.86 | 16.03 | 14.95 | 11.09 | 13.60 | 8.58 | 16.89 | 15.51 |
| Cluster-15348.69282 | 12.63 | 4.93 | 4.87 | 8.96 | 3.71 | 1.01 | 0.24 | 6.16 |
| Cluster-15348.69360 | 30.65 | 45.41 | 17.55 | 7.30 | 7.08 | 0.00 | 2.28 | 12.68 |
| Cluster-15348.70102 | 59.84 | 50.25 | 61.42 | 156.92 | 166.20 | 280.50 | 56.26 | 42.30 |
| Cluster-15348.70535 | 12.57 | 8.58 | 8.05 | 4.90 | 8.41 | 4.11 | 9.54 | 11.85 |
| Cluster-15348.70538 | 47.53 | 39.11 | 27.79 | 19.65 | 28.28 | 15.41 | 32.93 | 45.40 |
| Cluster-15348.71184 | 15.70 | 14.75 | 14.57 | 12.29 | 8.00 | 4.32 | 11.28 | 15.58 |
| Cluster-15348.71288 | 5.08 | 5.19 | 3.72 | 2.94 | 2.73 | 0.84 | 2.15 | 3.11 |
| Cluster-15348.71316 | 8.15 | 9.08 | 5.88 | 2.08 | 4.14 | 1.20 | 3.87 | 8.89 |
| Cluster-15348.71814 | 4.79 | 4.00 | 5.15 | 12.01 | 19.54 | 52.76 | 8.26 | 5.73 |
| Cluster-15348.72024 | 21.47 | 16.54 | 15.90 | 16.52 | 12.84 | 6.71 | 19.37 | 13.61 |
| Cluster-15348.72829 | 29.21 | 17.76 | 23.97 | 27.53 | 19.13 | 3.22 | 27.78 | 21.28 |
| Cluster-15348.73060 | 55.74 | 58.05 | 49.94 | 51.02 | 38.25 | 16.80 | 56.63 | 48.15 |
| Cluster-15348.73233 | 25.27 | 24.15 | 27.79 | 65.05 | 65.63 | 160.80 | 27.19 | 21.37 |
| Cluster-15348.73258 | 17.55 | 14.40 | 11.11 | 7.07 | 9.91 | 6.27 | 9.60 | 17.77 |
| Cluster-15348.73300 | 1.77 | 3.37 | 1.97 | 2.29 | 5.45 | 6.59 | 3.97 | 5.89 |
| Cluster-15348.74439 | 76.82 | 47.31 | 41.91 | 21.14 | 23.01 | 23.73 | 37.25 | 39.40 |
| Cluster-15348.74440 | 35.93 | 19.47 | 25.22 | 15.51 | 13.53 | 9.96 | 22.97 | 20.53 |
| Cluster-15348.74748 | 89.77 | 76.21 | 97.03 | 207.89 | 225.88 | 384.67 | 87.09 | 70.13 |
| Cluster-15348.75000 | 7.46 | 7.52 | 8.43 | 12.73 | 16.44 | 22.68 | 9.47 | 6.58 |
| Cluster-15348.75344 | 39.48 | 37.69 | 44.87 | 105.07 | 131.27 | 215.47 | 39.90 | 26.49 |
| Cluster-15348.75376 | 3.54 | 2.96 | 3.45 | 2.26 | 2.48 | 1.26 | 3.02 | 2.65 |
| Cluster-15348.75383 | 75.62 | 59.91 | 69.94 | 145.37 | 153.29 | 302.94 | 69.29 | 56.48 |
| Cluster-15348.75784 | 80.54 | 65.02 | 52.57 | 34.79 | 49.77 | 26.79 | 54.56 | 82.80 |
| Cluster-15348.76884 | 9.13 | 7.01 | 8.86 | 6.19 | 4.70 | 2.80 | 5.06 | 6.86 |
| Cluster-15348.76907 | 105.53 | 70.83 | 36.79 | 42.79 | 50.96 | 30.59 | 29.49 | 102.27 |
| Cluster-15348.77182 | 5.81 | 6.06 | 6.79 | 18.26 | 23.05 | 40.88 | 7.62 | 4.45 |
| Cluster-15348.77365 | 19.86 | 16.09 | 16.51 | 9.94 | 10.52 | 7.18 | 13.26 | 16.95 |
| Cluster-15348.77704 | 71.36 | 0.00 | 0.05 | 17.22 | 10.85 | 0.00 | 131.68 | 54.62 |
| Cluster-15348.78007 | 0.01 | 0.06 | 0.62 | 0.04 | 0.83 | 0.48 | 0.21 | 0.13 |
| Cluster-15348.78169 | 59.78 | 48.56 | 64.47 | 142.08 | 153.04 | 292.80 | 46.00 | 36.09 |
| Cluster-15348.78316 | 17.50 | 14.28 | 10.43 | 6.11 | 8.81 | 3.94 | 7.84 | 19.04 |
| Cluster-15348.78404 | 16.64 | 9.82 | 9.82 | 7.59 | 7.96 | 4.57 | 8.18 | 13.36 |
| Cluster-15348.78713 | 73.91 | 67.76 | 68.55 | 242.20 | 179.58 | 360.83 | 37.48 | 38.58 |
| Cluster-15348.79350 | 22.85 | 20.43 | 21.77 | 15.35 | 15.51 | 8.68 | 22.22 | 14.17 |
| Cluster-15348.79447 | 33.34 | 24.24 | 24.69 | 11.78 | 20.29 | 10.61 | 19.96 | 22.61 |
| Cluster-15348.79725 | 6.37 | 6.25 | 4.31 | 2.56 | 1.88 | 0.48 | 2.61 | 7.27 |
| Cluster-15348.79820 | 31.56 | 26.83 | 35.55 | 38.57 | 22.03 | 11.31 | 26.47 | 28.41 |
| Cluster-15348.79863 | 31.27 | 27.23 | 23.67 | 15.13 | 14.95 | 7.67 | 20.54 | 19.83 |
| Cluster-15348.79948 | 391.36 | 228.35 | 137.28 | 90.00 | 61.67 | 53.53 | 144.21 | 248.52 |
| Cluster-15348.80058 | 271.13 | 351.51 | 454.15 | 959.11 | 1224.29 | 1949.90 | 225.52 | 158.57 |
| Cluster-15348.80395 | 35.85 | 30.77 | 38.11 | 88.75 | 103.65 | 187.31 | 36.47 | 29.36 |
| Cluster-15348.80413 | 74.50 | 101.54 | 169.32 | 360.57 | 510.17 | 869.46 | 100.39 | 71.03 |
| Cluster-15348.80528 | 147.94 | 189.72 | 298.75 | 603.75 | 583.65 | 950.38 | 314.78 | 130.87 |
| Cluster-15348.80639 | 128.55 | 102.48 | 106.20 | 85.03 | 46.49 | 37.50 | 75.62 | 87.71 |
| Cluster-15348.80694 | 562.64 | 528.56 | 310.93 | 227.26 | 284.14 | 111.46 | 387.82 | 455.28 |
| Cluster-15348.80834 | 83.86 | 60.67 | 29.71 | 36.15 | 41.99 | 20.70 | 21.36 | 80.93 |
| Cluster-15348.80972 | 228.62 | 168.07 | 137.76 | 93.17 | 124.51 | 76.09 | 156.95 | 224.05 |
| Cluster-15348.81117 | 28.20 | 27.20 | 21.18 | 58.85 | 62.38 | 112.27 | 63.60 | 52.92 |
| Cluster-15348.81295 | 129.94 | 50.20 | 49.45 | 62.68 | 43.93 | 24.73 | 53.57 | 77.36 |
| Cluster-15348.81301 | 116.34 | 138.69 | 157.16 | 264.41 | 293.42 | 375.46 | 246.60 | 236.31 |
| Cluster-15348.81719 | 26.04 | 27.53 | 26.71 | 46.01 | 49.08 | 68.16 | 56.04 | 57.38 |
| Cluster-15348.81973 | 35.17 | 22.87 | 14.95 | 12.44 | 9.64 | 2.52 | 7.31 | 9.99 |
| Cluster-15348.82027 | 29.92 | 25.49 | 31.25 | 75.00 | 89.05 | 186.60 | 27.06 | 19.62 |
| Cluster-15348.82398 | 27.63 | 35.79 | 20.44 | 6.30 | 13.31 | 2.51 | 3.03 | 15.65 |
| Cluster-15348.82717 | 10.65 | 8.07 | 9.12 | 6.15 | 6.34 | 3.36 | 7.63 | 7.51 |
| Cluster-15348.83218 | 57.93 | 56.77 | 39.63 | 35.99 | 28.66 | 11.61 | 30.64 | 61.02 |
| Cluster-15348.83227 | 142.78 | 115.06 | 74.22 | 60.56 | 40.04 | 18.45 | 40.09 | 86.34 |
| Cluster-15348.83294 | 26.12 | 7.07 | 21.25 | 8.47 | 7.46 | 2.20 | 11.31 | 5.52 |
| Cluster-15348.83323 | 15.15 | 14.25 | 22.03 | 11.32 | 11.94 | 3.83 | 9.24 | 10.48 |
| Cluster-15348.83349 | 7.43 | 6.81 | 3.00 | 5.26 | 3.72 | 1.63 | 3.98 | 3.24 |
| Cluster-15348.85068 | 21.67 | 24.48 | 26.60 | 72.70 | 77.18 | 171.03 | 25.45 | 17.37 |
| Cluster-15348.85542 | 144.50 | 119.64 | 88.59 | 62.07 | 82.90 | 53.41 | 100.88 | 141.70 |
| Cluster-15348.85869 | 47.04 | 36.90 | 26.50 | 23.93 | 11.55 | 4.44 | 13.54 | 23.97 |
| Cluster-15348.85929 | 40.23 | 25.90 | 18.28 | 9.77 | 9.85 | 6.82 | 12.71 | 38.00 |
| Cluster-15348.86303 | 14.42 | 12.94 | 14.62 | 30.51 | 27.51 | 56.88 | 12.12 | 11.46 |
| Cluster-15348.86304 | 6.54 | 5.12 | 4.21 | 3.70 | 3.18 | 1.88 | 3.85 | 3.83 |
| Cluster-15348.86371 | 6.80 | 4.38 | 5.11 | 2.62 | 3.82 | 1.43 | 4.12 | 5.21 |
| Cluster-15348.86742 | 2.90 | 1.50 | 1.75 | 0.44 | 0.47 | 0.16 | 0.56 | 1.07 |
| Cluster-15348.87096 | 42.88 | 39.09 | 35.43 | 65.21 | 77.60 | 135.23 | 36.25 | 81.17 |
| Cluster-15348.87995 | 4.71 | 3.57 | 2.86 | 2.57 | 2.34 | 1.18 | 2.63 | 2.57 |
| Cluster-15348.89265 | 12.16 | 10.62 | 9.44 | 7.76 | 8.20 | 4.49 | 8.79 | 7.64 |
| Cluster-15348.89660 | 5.60 | 6.26 | 7.60 | 9.87 | 13.79 | 17.87 | 8.01 | 6.87 |
| Cluster-15348.90069 | 95.15 | 62.09 | 60.49 | 32.80 | 35.74 | 25.44 | 60.90 | 53.70 |
| Cluster-15348.90171 | 23.27 | 28.35 | 34.19 | 20.69 | 21.45 | 6.25 | 20.43 | 10.71 |
| Cluster-15348.90859 | 2.34 | 3.58 | 4.67 | 4.66 | 7.92 | 7.15 | 4.76 | 3.90 |
| Cluster-15348.91030 | 10.05 | 8.04 | 6.99 | 5.12 | 4.47 | 3.48 | 5.99 | 7.18 |
| Cluster-15348.91126 | 0.97 | 1.37 | 1.69 | 1.90 | 2.28 | 3.41 | 1.32 | 1.13 |
| Cluster-15348.91461 | 4.48 | 2.83 | 0.60 | 1.95 | 2.10 | 0.52 | 6.92 | 3.06 |
| Cluster-15348.91668 | 44.66 | 42.22 | 27.24 | 20.49 | 25.12 | 13.01 | 31.04 | 40.02 |
| Cluster-15348.92228 | 5.55 | 5.09 | 4.99 | 2.16 | 4.11 | 1.63 | 3.68 | 7.09 |
| Cluster-15348.94774 | 68.55 | 52.56 | 49.73 | 41.66 | 38.97 | 16.34 | 50.70 | 81.33 |
| Cluster-15348.94775 | 77.23 | 53.17 | 52.53 | 39.57 | 45.28 | 20.77 | 57.23 | 92.09 |
| Cluster-15348.94891 | 5.41 | 6.04 | 6.39 | 7.34 | 12.70 | 21.46 | 5.23 | 5.13 |
| Cluster-15348.96260 | 13.55 | 12.36 | 14.94 | 39.00 | 46.95 | 78.33 | 13.85 | 9.78 |
| Cluster-15348.96741 | 11.47 | 9.47 | 3.10 | 1.28 | 4.03 | 0.78 | 5.07 | 16.37 |
| Cluster-15348.96977 | 3.09 | 1.35 | 0.68 | 2.70 | 1.16 | 0.10 | 2.40 | 0.79 |
| Cluster-15348.99664 | 3.14 | 5.74 | 6.96 | 8.87 | 6.34 | 9.56 | 6.17 | 4.21 |
| Cluster-27689.0 | 1.54 | 0.32 | 0.18 | 0.09 | 0.07 | 0.00 | 0.00 | 0.00 |
| Cluster-30101.1 | 0.71 | 0.32 | 0.14 | 0.02 | 0.04 | 0.00 | 0.00 | 0.00 |
